# State of the Globe: Catheterizations Continue to Cultivate Urinary Infections

**DOI:** 10.4103/0974-777X.62869

**Published:** 2010

**Authors:** Ahmed Nasr

**Affiliations:** *Department of Obstetrics and Gynecology, Women's Health Center, Assiut University, Egypt*

Around the globe, over 100 million urinary catheters are being consumed annually, which translates into almost 200 being used every single minute.[1] By the time you end up reading this article, an extra 2000 catheters would have possibly been placed, a big fraction of which is being used unnecessarily or inappropriately. Since their first introduction way back in the 1300s, urinary catheters have been used quite liberally, with inadequate attention to catheter-associated morbidity (CAM). Almost a quarter of all hospitalized patients have indwelling urethral catheters. On one hand, catheters may serve clinicians to accurately monitor urine output following surgery or during an acute illness, relieve urinary retention, and assist in carrying out a variety of investigative procedures.[2] On the other hand, however, they are held responsible for iatrogenic catheter-associated urinary tract infections (CAUTI), which are indeed quite preventable. Little is the benefit of routine catheterization; immense are the consequences, challenges and complications. At any given time an estimated one out of every 25 patients in a given community has an indwelling urinary catheter.[3] Urinary catheters are a potential culprit in up to 80% of cases of nosocomial UTI, which clearly demonstrates how sizable the problem of routine catheterization really is.[4] Undoubtedly, the short- and long-term consequences of UTI are well known. Over and above, an indwelling urethral catheter can potentially prolong hospital stay, interfere with adequate ambulation, increase patient discomfort, retard early return to daily activities and certainly increase the cost of healthcare.[5]

The study published in this issue clearly shows that inappropriate use of indwelling urinary catheterization is prevalent, even in a tertiary care teaching hospital, and that patients in the medical emergency section and those suffering from urinary incontinence, especially females, are at particular risk. This warrants thorough evaluation prior to catheter placement to help reduce the burden of inappropriate catheterization to an absolute minimum.

From an obstetrician's perspective, empirical use of urinary catheterization is widely practiced during cesarean delivery worldwide as it is erroneously believed that its placement can improve surgical exposure of the lower uterine segment, minimize urinary bladder injury and avoid postoperative urinary retention.[6] However, a recently published prospective, multicenter, randomized controlled trial (RCT) addressing the safety, feasibility and benefits of carrying out elective cesarean delivery without routine indwelling urinary bladder catheterization has come to important conclusions.[7] The results of this trial have demonstrated that routine urinary catheterization during cesarean delivery in hemodynamically stable women is unwarranted. Avoidance of placing a urinary catheter has resulted in a significant reduction in the incidence of UTI. The volume of urine produced during cesarean did not produce a significant degree of distension, difficulties or interference during surgery. Women who were offered cesarean delivery without use of a urinary catheter experienced a significantly shorter mean ambulation time and hospital stay; the costs of surgery were also significantly less. Such women were generally pleased and satisfied with avoidance of catheter placement. All women who experienced a previous cesarean delivery with the use of an indwelling catheter were even more pleased and satisfied with nonuse of the catheter, with their experience to carry out cesarean without indwelling urinary catheter described by most as excellent.[7]

Despite infection control policies and procedures, CAUTI rates remain a significant problem in many tertiary centers; using identified risk factors, tailored intervention strategies should be implemented to reduce the rates of CAUTI.[8] Implementation of an intervention to judge appropriateness of indwelling urinary catheters may result in significant reductions in duration of catheterization and incidence of CAUTI.[9] Besides infection control, other factors come into play when choosing a catheter; those include ease of use, comfort and cost. There is a lack of evidence to state that incidence of UTI is affected by use of coated or uncoated catheters, single (sterile) or multiple use (clean) catheters, self-catheterization or catheterization by others, or by any other strategy. The current research evidence is weak and design issues are significant.[10] However, a variety of techniques have been improvised to help reduce the risk of CAUTI. These include introduction of antiseptic impregnated catheters and antibiotic impregnated catheters. Currently available evidence suggests that short-term use of silver alloy catheters and those impregnated with antibiotics reduces the risk of CAUTI. Use of such catheters may be promising and appealing; however, further economic evaluation is required to confirm their cost-effectiveness.[11] There is suggestive but inconclusive evidence of a benefit from midnight removal of indwelling urethral catheters.[2] There is also weak evidence that antibiotic prophylaxis, rather than giving antibiotics when clinically indicated, reduces the rate of symptomatic UTI.[12]

Pragmatic use of urinary catheters is to be envisaged as an irrational attitude of negligence, ignorance, habituation or simply indifference. In spite of the aforementioned facts, undue and inappropriate catheter use continues to prevail.[1] Given the preponderance of such practice, or more accurately ‘malpractice’, every possible effort should be devoted to change attitudes towards routine catheterization in an earnest endeavor to curtail CAM. Time has now come, more than ever before, to make a real change of thought in the eon of evidence-based medicine (EBM). In fact the stage is set for such rightful thinking, since the duration of urinary catheterization and CAM were both significantly reduced when physicians were merely reminded to remove unnecessary urinary catheters. A urinary catheter should no longer be considered ‘routine’ and such a word should not be part of daily medical parlance. Selective use of catheters is a policy that should be praised and encouraged. This is particularly pertinent to developing nations, where any reduction in the incidence of UTI definitely has substantial repercussions on the overall quality of healthcare.

Moving on from theory to practice, physicians are far from reaching a unanimous agreement about catheter use. A competent doctor is expected to show a great deal of flexibility, versatility and above all wisdom, when deciding to place a catheter. It has indeed been my personal experience over the last two decades not to take a hurried decision, but rather consider catheterization a last resort. I do recommend that every one of us asks himself/herself one important question: is a catheter inevitably needed, or could we do well without? If so, should it be placed right now, and for how long?
